# Case report of a central venous access device-associated thrombosis with aortic embolism in a preterm infant

**DOI:** 10.1186/s12887-016-0691-x

**Published:** 2016-09-06

**Authors:** Marlene Biermayr, Barbara Brunner, Kathrin Maurer, Rudolf Trawoeger, Ursula Kiechl-Kohlendorfer, Vera Neubauer

**Affiliations:** 1 Department of Paediatrics II, Neonatology, Medical University of Innsbruck, Anichstrasse 35, 6020 Innsbruck, Austria; 2 Department of Radiology, Medical University of Innsbruck, Innsbruck, Austria

**Keywords:** Preterm, Central venous access device, Thrombosis, Aortic embolism, Paradoxical stroke, Case report

## Abstract

**Background:**

Thrombosis in neonates is commonly a central venous access device (CVAD) associated complication. Furthermore, a patent foramen ovale (PFO) is frequently seen in preterm infants. Even though a coincidence of both is not unusual, detaching of the thrombus and organisation of an aortic embolism has not been described until now. Treatment recommendations of CVAD-associated thrombosis in neonates do not consider frequently seen complications of preterm infants e.g. intraventricular haemorrhage.

This is the first case of a very preterm infant with pre-existing intraventricular haemorrhage, who developed a CVAD-associated thrombosis and thromboembolic complications.

**Case presentation:**

The authors report on a very preterm girl with a pre-existing intraventricular haemorrhage and a CVAD-associated thrombus that, after removal of the CVAD, led to assumed pulmonary embolism and to an extended aortic embolism with consequent cerebral stroke. The girl was treated with unfractionated heparin (UFH) for about 50 days. During the further in-hospital stay the girl developed a mild bronchopulmonary dysplasia. Follow-up revealed clinical signs of cerebral palsy.

**Conclusion:**

Even though preterm infants are often diagnosed with a PFO which constitutes the risk for paradoxical embolism, such complications do not occur frequently due to the physiological heart pressure proportion. Nevertheless, it is important to monitor vital parameters and cerebral perfusion after removing a CVAD with confirmed associated thrombosis, because thromboembolic complications are possible. If practicable, patients with a confirmed CVAD-associated thrombosis should be anticoagulated before removing the CVAD. However, in our patient it was rational to remove the CVAD without prior anticoagulation due to the pre-existing intraventricular haemorrhage.

There are various treatment recommendations for thrombosis or embolism in infants. However, there are no clear recommendations in very preterm infants with a high risk of cerebral bleeding respectively a pre-existing intraventricular haemorrhage. We decided to treat our patient with unfractionated heparin until the affected vessels were recanalised.

Finally, it remains a case-by-case decision how to treat CVAD-associated thrombosis and consequent embolism depending on the patient’s medical history.

## Background

Central venous access devices (CVAD) are a known risk factor for thrombosis in neonates. Furthermore, many preterm infants are diagnosed with a patent foramen ovale (PFO). Even though the combination of both is common in preterm neonates, development of pulmonary complications and paradoxical aortic embolism has not been described yet.

Existing guidelines for the treatment of thrombotic or thromboembolic complications do not consider the gestational age of the affected child and thereby disregard prevalent complications in relation to immaturity, such as intraventricular haemorrhage (IVH) [1]. This is a limiting factor in the applicability of recommended therapeutic measures for very preterm infants.

In this report we present the first case of a preterm infant with a pre-existing IVH with periventricular venous haemorrhagic infarction (PVHI), who developed a CVAD-associated thrombosis with thromboembolic complications after removal of the CVAD. The patient suffered from an extensive aortic embolism, which led to seizures caused by ischaemic brain damage due to an occlusion of large brain supplying arteries and, or, cerebral embolism. In addition, the patient presented with symptoms of pulmonary embolism.

Moreover, we discuss treatment recommendations of thrombotic complications and their applicability in sick preterm neonates.

## Case presentation

The girl was born at the age of 30 weeks of gestation by a Caesarean section due to pre-eclampsia of the mother. Birth weight was appropriate for gestational age, umbilical pH was 7,27 and Apgar score was 6-8-9. Because of respiratory distress she was treated with surfactant and extubated on nasal continuous positive airway pressure ventilation after 8 h. A cranial ultrasound on postnatal day three revealed a right-sided IVH/PVHI.

**Fig. 1:**
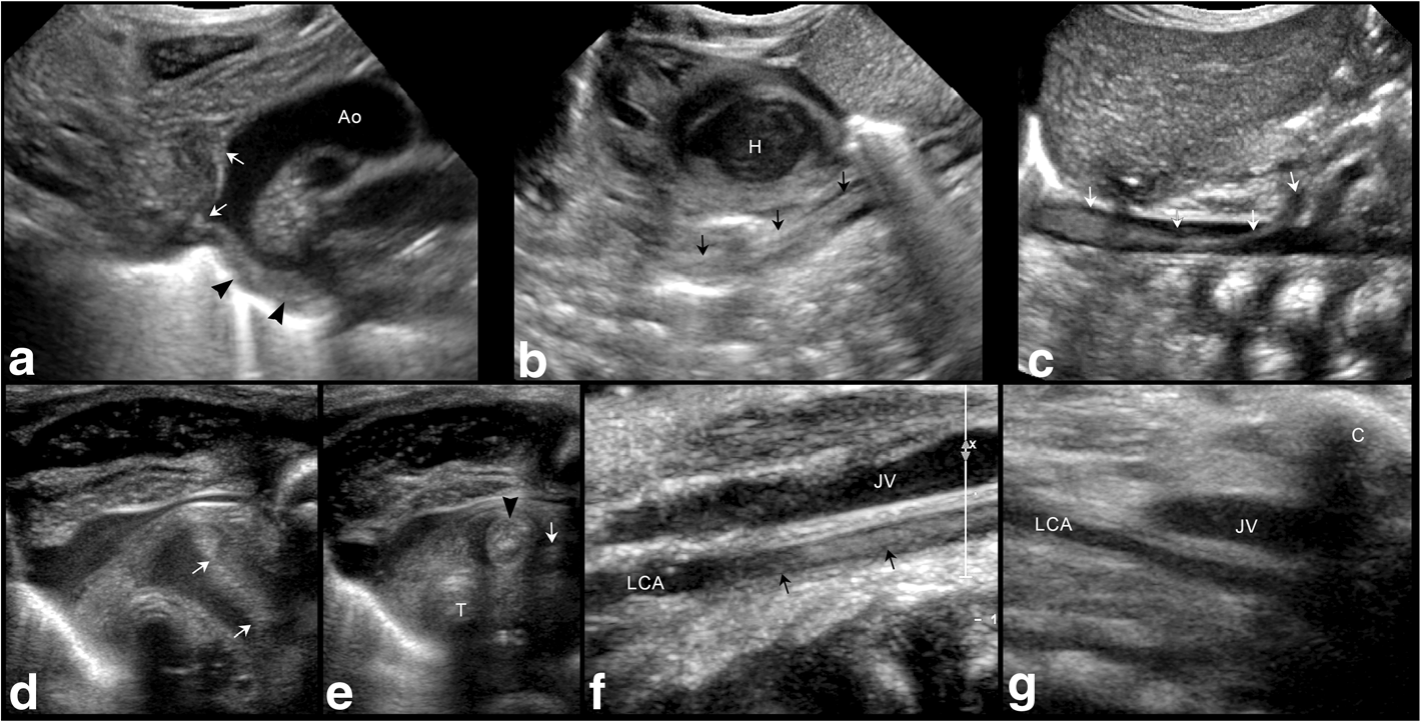
Vascular ultrasound. *Upper row* showing sagittal images of the embolus in the **a** aortic arch (*arrowheads*) with its extention into the innominate artery and the left carotid artery (*white arrows*); **b** descending aorta (*arrows*) and **c** abdominal aorta (*arrows*) with its end just above the celiac trunk. *Lower row* showing axial images of the upper mediastinum **d** with the embolus in the aortic arch (*white arrows*) and **e** the innominate artery (*arrowhead*) and in the left carotid artery (*arrow*); sagittal images of the neck **f** with the embolus within the supraclavicular part of the left carotid artery (*arrows*), **g** which is no longer visible 2 days later. Ao – aorta, H – heart, T – trachea, JV – jugular vein, LCA – left carotid artery, C - clavicula

During a routine echocardiography on postnatal day six, a PFO but no other morphologic or functional abnormality was observed. Additionally, a large thrombus (3,5 mm × 8,0 mm) on the tip of the peripherally inserted central venous catheter (PICC (premicath©, Vygon, Germany), which was inserted from the right ankle as part of the initial care, floating next to the atrial septum was detected. The catheter was removed immediately. Twelve hours later the girl’s condition suddenly deteriorated. She showed fits, skin colour was pale and her limbs were cold. Furthermore, she presented with respiratory insufficiency. In the course of intubation, blood, which apparently originated from a lung haemorrhage, was seen. Ultrasound examination showed a long embolus in the aortic arch, which extended to the innominate artery, the left carotid artery and to the descending aorta to just above the celiac trunk (Fig. 1). Additionally to the pre-existing IVH/PVHI an ischaemic infarction of the majority of the left hemisphere was seen. We assumed that the thrombus not only shifted to the aorta but also to the pulmonary artery and thereby caused a pulmonary embolism that led to the lung haemorrhage, as echocardiography showed no reopening of the ductus arteriosus.

Regarding recurrent sanguinary secretions in the ventilation tube heparin treatment was started at a low dosage (5 units/kg/hour) and was progressively increased, depending on the partial Thromboplastin time (PTT), signs of haemorrhage and size of the thrombus during the next 25 days up to a maximum of 15 units/kg/hour. Besides, a supportive treatment with repeated administration of fresh frozen plasma (FFP) was started. Nonetheless, Antithrombin III levels were low (40 % after first administration of FFP), therefore substitution was started to reach levels >100 % to improve heparin effectiveness. Protein C levels were normal. Unfractionated heparin (UFH) was administered for 51 days (48 days >10 units/kg/hour). After this period the innominate artery was recanalised and the blood flow in the left carotid artery was normal. Detailed diagnostic work-up to exclude causes for thrombophilia did not reveal any abnormality neither in the infant nor in her mother.

**Fig. 2:**
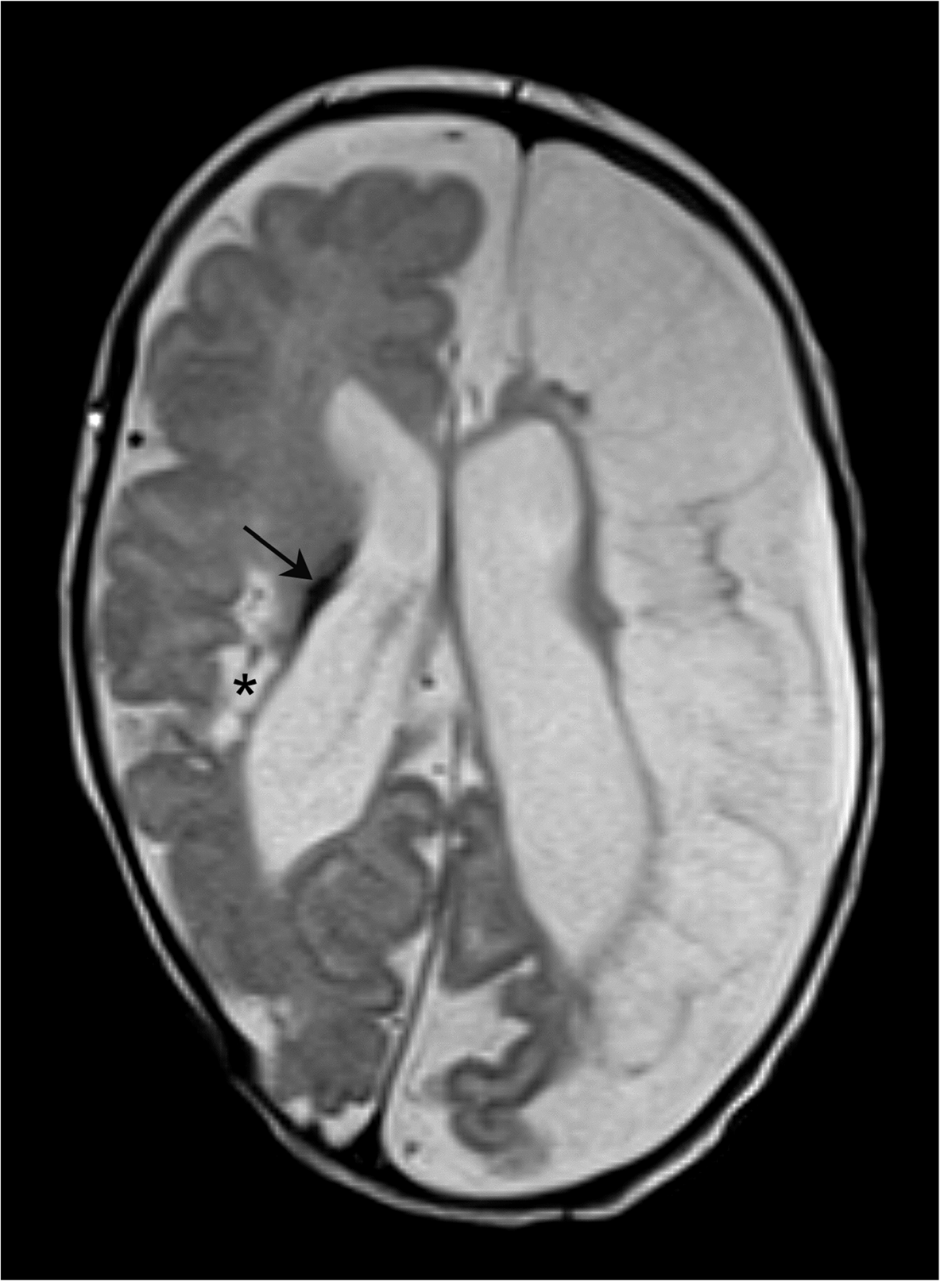
Cerebral MRI scan at term equivalent age showing a postischaemic cystic encephalomalacia of the left hemisphere and the intraventricular haemorrhage (*arrow*) with posthaemorrhagic cysts (*) on the right side with a consequent e vacuo dilatation of the lateral ventricles

Extubation was possible 4 days after the initial event. During the further in-hospital stay the girl developed a mild bronchopulmonary dysplasia but no further pulmonary complications. She exhibited persistent muscular hypotonia and pronounced myoclonuses, but no persisting seizures. She was discharged at a postmenstrual age of 39 weeks. A cerebral magnetic resonance imaging (MRI) at term equivalent age showed a postischaemic cystic encephalomalacia of the left hemisphere and posthaemorrhagic cysts on the right side (Fig. 2). Follow-up with a corrected age of 3 months revealed a hypertonic lower extremity and functional deficits, especially on the left side. Moreover, the patient had no fidgety movements in the general movements assessment. These findings are highly associated with the development of cerebral palsy [2].

## Conclusion

To the best of our knowledge this is the first case of a preterm infant with an IVH/PVHI and a PFO, who developed a CVAD-associated thrombosis and subsequently suffered from assumed pulmonary embolism and aortic embolism with extensive cerebral ischaemic infarction.

As mentioned above, many preterm infants have a PFO and it is known that a venous or cardiac thrombus may cause a paradoxical stroke and, or, an arterial embolism [3–6]. Even though our patient exhibited a thrombus in the right atrium and a PFO was observed during the echocardiography, an aortic embolism was not to be expected due to the physiological heart pressure proportion. Furthermore, it is remarkable that even though emboli detached and led to an assumed pulmonary embolism and to a cerebral stroke, there were no further clinical complications due to emboli after the initial event.

A Canadian study reported that almost 90 % of all thromboses in newborns were related to a CVAD [7]. According to recommendations for the maintenance of CVAD patency, we administer 0.5 units/kg/hour UFH to all infants with PICC in our neonatal intensive care unit [1]. A Cochrane Review showed that this heparin prophylaxis reduces the risk of PICC occlusion, but not the risk of PICC-associated thrombosis [8]. In the case of a confirmed CVAD-associated venous thrombosis, Monagle et al. suggest to remove the CVAD (grade 1B) and recommend anticoagulation with UFH or low molecular weight heparin (LMWH) for three to five days (grade 2C). They also regard the case of right atrial thrombosis related to a CVAD and suggest to remove the catheter with or without anticoagulation, depending on individual risk factors [1]. At that time-point treatment with heparin at dosages affecting the PTT in our patient was hazardous due to the IVH/PVHI, for which reason the catheter was removed without prior anticoagulation. After PICC removal and demarcation of the embolus in the aorta and adjacent large arterial vessels, a thrombolysis was not feasible due to the cerebral and pulmonary haemorrhagic complications. A thrombectomy was considered as not feasible, because of the small dimensions of the vascular system and the necessity of post-interventional effective anticoagulation. After careful appraisal of the benefit risk ratio in the situation of extensive aortic embolism with partial occlusion of major brain supplying arteries with concomitant acute cerebral and pulmonary haemorrhage, we started an intravenous UFH treatment at 5 units/kg/hour based on the suggestion of Monagle et al. to treat “neonates with a first acute ischemic stroke and a documented cardioembolic source” with heparin [1]. Under permanent monitoring of haemorrhagic complications the dosage was increased with extreme caution. Fortunately no further bleedings occurred.

After initial anticoagulation, Monagle et al. suggest further treatment with subcutaneous LMWH for a total duration of 6 weeks to 3 months [1]. A daily subcutaneous drug administration is challenging in infants with low weight and little subcutaneous fat tissue. Therefore, we decided to treat our patient with intravenous UFH until the affected vessels were recanalised after about 50 days.

In conclusion, removal of the PICC without prior anticoagulation was rational in this case. Nevertheless, if possible, patients with a confirmed thrombosis should be anticoagulated before removing a CVAD. After removal, monitoring of vital parameters and cerebral perfusion should be performed because, even though these complications are uncommon, pulmonary embolism is possible and a PFO constitutes a risk for paradoxical embolism. Obviously, it is not possible to give a general applicable recommendation for treating CVAD-associated thrombosis or aortic embolism in preterm infants. Thus, it remains a case-by-case decision depending on the patient’s condition, thrombophilic factors and previous complications such as (intracerebral) haemorrhage.

## Abbreviations

CVAD: Central venous access device
FFP: Fresh frozen plasma
fig.: Figure
IVH: Intraventricular haemorrhage
LMWH: Low molecular weight heparin
MRI: Magnetic resonance imaging
PFO: Patent foramen ovale
PICC: Peripherally inserted central catheter
PTT: Partial thromboplastin time
PVHI: Periventricular venous haemorrhagic infarction
UFH: Unfractionated heparin

## References

[1] Monagle P, Chan AKC, Goldenberg NA, Ichord RN, Journeycake JM, Nowak-Göttl U (2012). Antithrombotic Therapy in Neonates and Children. Chest J.

[2] Einspieler C, Prechtl HFR (2005). Prechtl’s assessment of general movements: A diagnostic tool for the functional assessment of the young nervous system. Ment Retard Dev Disabil Res Rev.

[3] Parker MJ, Joubert GI, Levin SD (2002). Portal vein thrombosis causing neonatal cerebral infarction. Arch Dis Child Fetal Neonatal Ed.

[4] Filippi L, Palermo L, Pezzati M, Dani C, Matteini M, De Cristofaro MT (2004). Paradoxical embolism in a preterm infant. Dev Med Child Neurol.

[5] Beattie LM, Butler SJ, Goudie DE (2006). Pathways of neonatal stroke and subclavian steal syndrome. Arch Dis Child Fetal Neonatal Ed.

[6] Amlie-Lefond CM, Basir MA, Franciosi RA (2008). Fatal Neonatal Stroke From a Prenatal Cardiac Thrombus. Pediatr Neurol.

[7] Schmidt B, Andrew M (1995). Neonatal thrombosis: report of a prospective Canadian and international registry. Pediatrics.

[8] Shah PS, Shah VS, Shah PS (2008). Continuous heparin infusion to prevent thrombosis and catheter occlusion in neonates with peripherally placed percutaneous central venous catheters. Cochrane Database Syst. Rev. [Internet].

